# Current Methods of Post-Translational Modification Analysis and Their Applications in Blood Cancers

**DOI:** 10.3390/cancers13081930

**Published:** 2021-04-16

**Authors:** Katie Dunphy, Paul Dowling, Despina Bazou, Peter O’Gorman

**Affiliations:** 1 Department of Biology, National University of Ireland, W23 F2K8 Maynooth, Ireland; katie.dunphy.2015@mumail.ie (K.D.); paul.dowling@mu.ie (P.D.); 2 Department of Haematology, Mater Misericordiae University Hospital, D07 WKW8 Dublin, Ireland; despina.bazou@ucd.ie

**Keywords:** post-translational modifications, blood cancer, phosphorylation, ubiquitination, sumoylation, acetylation, multiple myeloma, leukemia, lymphoma, myeloproliferative neoplasms

## Abstract

**Simple Summary:**

This review discusses current techniques used to study post-translational protein modifications (PTMs) and their contribution to blood cancer research. Blood cancer is an umbrella term for cancers that affect blood cells and organs of the hematopoietic system, accounting for approximately 10% of all cancer diagnoses. PTMs are crucial for the normal functioning of cells via the regulation of protein structure, function, and localization. Mass spectrometry and antibody-based techniques are powerful analytical tools used for the detection and quantitation of PTMs such as phosphorylation. The application of these techniques in blood cancer research facilitates the identification of abnormal PTMs that contribute to cancer development and progression by promoting cancer cell growth, survival, and invasion. Many FDA-approved blood cancer treatments exert their anti-cancer effects by targeting protein modifications, thus emphasizing the importance of PTM-focused research in the identification of novel biomarkers and therapeutic targets to improve blood cancer survival outcomes.

**Abstract:**

Post-translational modifications (PTMs) add a layer of complexity to the proteome through the addition of biochemical moieties to specific residues of proteins, altering their structure, function and/or localization. Mass spectrometry (MS)-based techniques are at the forefront of PTM analysis due to their ability to detect large numbers of modified proteins with a high level of sensitivity and specificity. The low stoichiometry of modified peptides means fractionation and enrichment techniques are often performed prior to MS to improve detection yields. Immuno-based techniques remain popular, with improvements in the quality of commercially available modification-specific antibodies facilitating the detection of modified proteins with high affinity. PTM-focused studies on blood cancers have provided information on altered cellular processes, including cell signaling, apoptosis and transcriptional regulation, that contribute to the malignant phenotype. Furthermore, the mechanism of action of many blood cancer therapies, such as kinase inhibitors, involves inhibiting or modulating protein modifications. Continued optimization of protocols and techniques for PTM analysis in blood cancer will undoubtedly lead to novel insights into mechanisms of malignant transformation, proliferation, and survival, in addition to the identification of novel biomarkers and therapeutic targets. This review discusses techniques used for PTM analysis and their applications in blood cancer research.

## 1. Introduction

**Figure 1:**
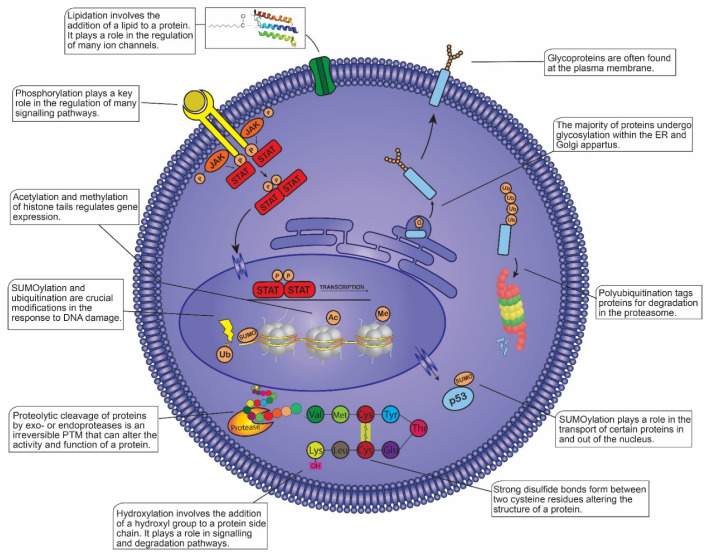
Post-translational modifications (PTMs) within the mammalian cell. This figure illustrates some of the most well-known PTMs and their functions within the cell. PTMs are found throughout the cell from the plasma membrane to the nucleosomes present within the nucleus. PTMs play crucial roles in almost all cellular processes including the cell cycle, degradation, apoptosis, cell signaling, transcription, etc. Different proteins modified by the same PTM will not always yield the same response, demonstrating the diverse functions of PTMs within the cell. JAK, Janus kinase; STAT, signal transducer and activator of transcription; P, phosphate group; G, glycosyl group; Ub, ubiquitin; SUMO, small ubiquitin-like modifier; Ac, acetyl group; Me, methyl group; Val, valine; Met, methionine; Cys, cysteine; Tyr, tyrosine; Glu, glutamic acid; Lys, lysine.

The significance of proteome-focused studies in characterizing cellular phenotypes and disease mechanisms, in addition to discovering novel biomarkers and therapeutic targets, has been recognized in recent decades [1]. Proteomics involves the characterization and mapping of the proteome, a highly complex task due to temporal and spatial fluctuations in protein expression depending on the cellular environment. The post-translational modification (PTM) of proteins, a biochemical process that modifies the structure and function of a given protein, further enhances the complexity of the proteome [2]. Over 200 PTMs have been identified, many of which play vital roles in the regulation of various cellular processes such as cell growth, metabolism, differentiation, and apoptosis (Figure 1). Dysregulated PTMs alter the normal functioning of these processes which can contribute to the development and/or progression of disease, thus illustrating the need to identify and define primary PTM events associated with specific diseases such as blood cancers [3].

Hematological malignancies arise from blood or bone marrow cells that acquire genetic abnormalities resulting in uncontrolled proliferation, resisting cell death, and evading the immune response [4]. The incidence of hematological malignancies, which comprises a broad range of cancers including leukemias, lymphomas, multiple myeloma, myeloproliferative neoplasms (MPNs) and myelodysplastic syndromes (MDS), continues to rise, although advances in treatment have led to an increase in the five-year survival rate of many of these cancers [5]. In this review, we provide a comprehensive overview of current analytical techniques employed in the analysis of post-translational modifications and emphasize the contribution of these techniques to our current understanding of disease mechanisms associated with blood cancers. We highlight the central role of PTMs in the development of blood cancer therapeutics and consider the promising prospect of future improvements in the sensitivity and specificity of PTM-focused techniques to expedite our understanding of hematological malignancies and offer novel biological markers and therapeutic targets to augment the current arsenal of therapeutics available for the treatment of blood cancers.

## 2. Analytical Techniques in Post-Translational Modification Analysis

**Figure 2:**
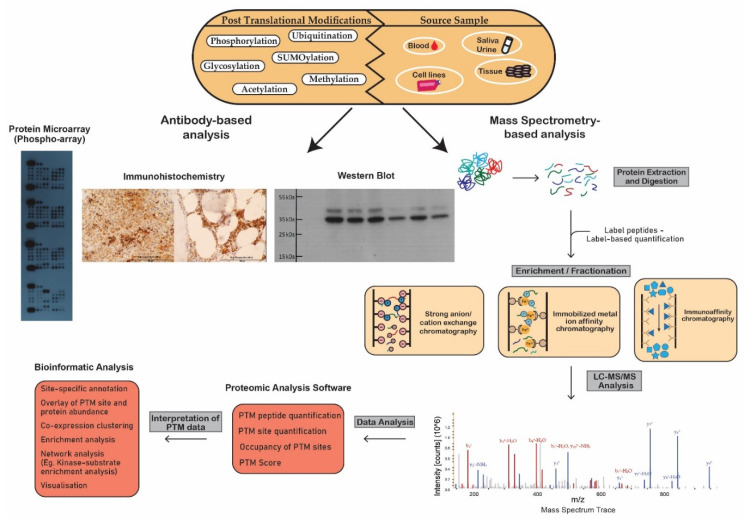
Schematic workflow illustrating analytical techniques used in the analysis of post-translational modifications. Proteomic techniques used for PTM analysis can be divided into antibody-based analysis or mass spectrometry (MS)-based analysis. Protein microarrays, immunohistochemistry and Western blot analysis demonstrate widespread popularity as efficient tools for antibody-based PTM analysis. MS-based analysis is a powerful technique in the study of PTMs. This figure outlines the steps involved in the preparation of crude samples for MS and the transformation of MS data into interpretable results.

The most powerful analytical technique applied in proteomic studies is mass spectrometry (MS), which facilitates large-scale, highly specific, quantitative profiling of proteins and post-translationally modified proteins. Other techniques often used to study the proteome include flow cytometry, protein microarrays, Western blotting, and enzyme-linked immunosorbent assays (ELISA) (Figure 2) [1]. Additional experimental steps are often required to optimize proteomic techniques for PTM analysis due to the substoichiometric, transient and labile nature of PTMs which hinders the maintenance and detection of modifications during analysis [2]. To overcome the issue of low stoichiometry, enrichment of the PTM of interest is often performed prior to MS analysis. Modification-specific enrichment techniques such as ion exchange chromatography, immobilized metal ion affinity chromatography and immunoaffinity chromatography, separate modified proteins/peptides from their unmodified counterparts, reduce the complexity of the sample and increase the efficiency and reliability of the analysis (Figure 2) [3].

Highly sensitive mass spectrometers with a high resolution, such as the Fourier transform (FT)-based mass spectrometers, are required for efficient PTM analysis. The “bottom-up” proteomics approach, typically used in PTM analysis, involves the cleavage of proteins in a biological sample by a protease, usually trypsin, prior to MS [6]. Sample preparation techniques, such as filter-aided sample preparation (FASP), separate peptides from undigested material in cell lysates [7]. Most MS-based proteomic protocols couple liquid chromatography with tandem MS (LC–MS/MS) to separate peptides before identification and quantitation by MS/MS. Following separation, the LC eluent is transferred to the ion source of the mass spectrometer where peptides are ionized, often via protonation [8]. High ionization efficiency enhances the sensitivity of the analysis; however, the efficiency of this step can be affected by the chemical nature of peptides being studied [9]. The dominant ionization technologies are matrix-assisted laser desorption/ionization (MALDI), electro-spray ionization (ESI) and atmospheric pressure chemical ionization (APCI) [10]. Following ionization, the peptide precursor ions are fragmented resulting in product ions that are subsequently characterized based on their mass-to-charge (m/z) ratios and relative abundance to produce MS/MS spectra (Figure 2) [9]. Popular fragmentation techniques include collision-induced dissociation (CID) and high-energy collision dissociation (HCD). However, limitations, such as the loss of labile PTMs, mean alternative fragmentation methods, including electron capture/transfer dissociation (ECD/ETD), have been developed and incorporated into MS-based PTM analyses [11]. During CID and HCD, the collision of molecular ions results in cleavage of the amide bond in peptides whereas ETD involves the transfer of an electron to multiply protonated peptides resulting in the cleavage of the peptide backbone [12]. The more recently developed fragmentation methods, EThcD, which combines ETD and HCD, and activated ion ETD (AI-ETD) demonstrate great progress in combatting the challenges presented by conventional fragmentation techniques in PTMomics, such as uncertain site localizations [13,14,15]. The MS/MS spectra obtained during LC–MS/MS are matched to theoretical MS/MS spectra available on databases for peptide and PTM identification. Bioinformatic analysis, using algorithms that predict the probability of a PTM being present such as Ascore, PTMScore, PhosphoRS and Mascot Delta Score, are used to determine the validity of modification sites detected during LC–MS/MS [16].

MS-based quantitation of proteins is typically divided into label-based and label-free techniques. Labeling peptides using differential mass tags, such as tandem mass tag (TMT) or isobaric tag for relative and absolute quantification (iTRAQ), allows for the comparison and multiplexing of multiple samples in a single MS run [17]. These isobaric labeling technologies involve the labelling of target proteins/peptides in different biological samples with tags made up of a reactive group, a mass normalization group, and a reporter group that have an identical overall mass but differ in the mass of the reporter group and mass normalization group. The reactive group facilitates the mass labeling of peptides. Fragmentation during tandem MS cleaves the reporter group, revealing reporter ions of variable molecular weights corresponding to the peptides present in the individual biological samples [18]. The in vitro labeling technique, stable-isotope labeling of amino acids in cell culture (SILAC), involves the labeling of peptides by growing an organism in cultures containing light or heavy stable isotope-containing amino acids (^13^C, ^15^N), typically lysine and arginine, which are subsequently metabolically incorporated into proteins. During MS analysis, source samples can be easily differentiated by the isotopic mass shifts between the different isotopically labeled samples [19]. Subsequently, this technique has been adapted to facilitate the labelling of specific PTMs. For example, ^13^C-glucose and D_3_-acetate, ^13^CD_3_-methionine; and γ-^18^O_4_-labeled ATP are added to cell culture media to label acetylation, methylation, and phosphorylation, respectively [20,21,22]. Label-free quantitation (LFQ) is a commonly used method of quantitation which refers to the use of peak intensity analysis and spectral counting for quantitation [23]. The results of qualitative or quantitative studies can undergo downstream statistical analysis to draw conclusions from the data through statistical tests, such as t-tests or ANOVAs, and/or visualize the data by creating networks, plots, and graphs [24].

A substantial effort has been made in recent years to develop specific antibodies for PTM analysis. However, low binding affinity is common among available antibodies due to the minuteness of PTM motifs, similarities in the chemical structure of certain PTMs, poor antigenicity and other difficulties in antibody generation [3]. Pan-PTM-specific antibodies are often applied for immunoaffinity enrichment prior to LC–MS/MS as well as Western blotting, protein microarrays, immunohistochemistry, and flow cytometry. Antibodies against specific modification sites known to play an important biological role are also available for certain modifications including phosphorylation, methylation, and acetylation [25,26,27]. Other PTMs face difficulties in site-specific antibody generation due to the size or transience of the PTM (e.g., ubiquitination); or lack sufficient evidence of site occupancy to invest in antibody generation [28].

Flow cytometry (FCM) is a scientific technique used to measure the physical and biochemical characteristics of cells, including size, internal complexity, and cellular components such as DNA, RNA, and specific proteins on the cell surface or intracellularly. FCM has gained prominence in recent years and is now routinely used in basic research and clinical practice, particularly in the oncology and immunology settings. FCM is an extremely sensitive technique, capable of detecting an extremely small number of cancer cells within a dense cellular network. This technique also provides a quick turnaround time, with the ability to measure a significant number of parameters in thousands of cells in a matter of seconds. The detection of surface antigens by FCM can be used in real time to physically sort cells into specific populations, a process known as cell sorting or fluorescence-activated cell sorting (FACS). This is particularly useful for the analysis of hematological malignancies with immunophenotyping by FCM often used in the clinical diagnosis and subclassification of leukemias and lymphomas. Once the cells of interest are selected, qualitative information may be obtained by measuring fluorescence intensity for specific antigens, for example acute leukemia (CD45), chronic lymphocytic leukemia (CD19/CD5), and Non-Hodgkin’s lymphoma (CD20) [29,30]. FCM-based minimal residual disease (MRD) assays are powerful diagnostic and disease monitoring tools in multiple types of blood cancers. By detecting low-frequency malignant cells (ratio as low as 1:10000 compared to normal cells), this approach is critical in the clinical management of patients [31]. As MRD status appears to correlate with patient outcomes in some hematological malignancies, increasing the sensitivity and robustness of MRD assays analyzed by FCM is currently an active area of research. Ongoing improvements in the quality of PTM site-specific antibodies will strengthen FCM as a promising method for characterization of cellular activities and signal transduction pathways in hematological malignancies in research and clinical settings. When designing a PTM-focused experiment, researchers must consider additional sample preparation steps that may be required to analyze modifications on proteins from specific cellular components, such as the cell surface, extracellular vesicles, and the nucleus. Histone proteins represent a well-known target for PTM-focused analyses as they are extensively modified by various PTMs to facilitate dynamic genomic organization and transcriptional regulation. PTMs found on histones include methylation, acetylation and ubiquitination on lysine residues, phosphorylation on serine, threonine, and tyrosine residues and/or citrullination on arginine residues [32]. Histone PTMs are routinely detected through site-specific antibodies, as demonstrated recently by Xu and co-workers [33] investigating histone H3 lysine 36 dimethylation (H3K36me2) levels in myeloma cells under normoxia or hypoxia conditions using Western blotting [33]. By detecting both the protein and site-specific modification abundance levels using precise antibodies, the degree of histone modification can be quantified. However, in the past, some problems with respect to anti-PTM histone antibody quality surfaced, facilitating the need to develop alternative platforms [34]. Mass spectrometry has proven to be an invaluable research platform in identifying and quantifying histone PTMs. Several histone enrichment methods are available prior to analysis, including chromatin affinity purification with MS (ChAP–MS), an approach that facilitates the isolation of targeted chromatin sections for the identification of histone PTMs [35]. Another isolation method involves the use of acidic or high-salt conditions to extract histones from cell lysates [32]. Due to the high ratio of the basic amino acids, arginine and lysine, in histones, tryptic digestion generates short peptides (2–4 amino acids) that are incompatible with LC–MS/MS analysis. Thus, chemical derivatization, often via propionylation, of lysine residues has been implemented to inhibit cleavage after lysine, resulting in peptides with lengths compatible with LC–MS/MS [36]. Detection of histone PTMs from MS/MS spectra is accomplished by detecting mass shifts corresponding to the PTM of interest, as described in the sections below, to confirm the presence of the modification. Determining the function of histone PTMs often involves investigating interacting partners, in particular the suite of associated enzymes responsible for the regulation of site-specific modifications. Crosslinking mass spectrometry (XL–MS) has emerged as a powerful approach to elucidate protein–protein complexes by mass spectrometry, with applications for structural mechanism discovery involving histone activity [37].

### 2.1. Phosphorylation

Phosphorylation refers to the addition of a phosphoryl group to an amino acid residue, typically serine, threonine or tyrosine, resulting in the formation of a phosphodiester bond [38]. It is a reversible modification catalyzed by kinases and reversed by phosphatases. Phosphorylation is the most extensively studied PTM due to its major role in many cellular processes including cell signaling, transcriptional regulation, protein synthesis and degradation, metabolic pathways, and many others [39,40,41,42,43]. It is postulated that more than 90% of the human proteome is phosphorylated under certain conditions [38]. Unsurprisingly, abnormal phospho-signaling contributes to the development or exacerbation of a variety of diseases; with phosphoproteomic analysis revealing many biomarkers of disease and therapeutic targets [44]. For example, tyrosine kinase inhibitors (TKIs) are now a major class of therapeutics used in the treatment of many forms of cancer including chronic myeloid leukemia, breast, and lung cancers [45].

Despite widespread research focused on phosphorylation, the identification and quantitation of phosphorylated residues remains a challenge due to substoichiometry, the ability of a protein to be phosphorylated on several residues and difficulties in selective phosphopeptide isolation [46,47]. Conventional electrophoresis-based methods may be employed to analyze phosphoproteins; however, low throughput and reproducibility reduces the robustness of this technique [48]. Two-Dimensional Gel Electrophoresis (2D-PAGE) involves the separation of proteins based on their mass and isoelectric point and can be used as a method of protein separation prior to MS analysis. Different proteoforms are separated and quantitatively compared through differences in staining intensity after visualization using highly specific dyes. The fluorescent dye Pro-Q Diamond is typically used for phosphopeptide analysis [49,50]. Nowadays, the more advanced electrophoresis and antibody-based method, Western blotting, is commonly used for phosphoproteomic analysis. Following electrophoresis, the separated proteins are exposed to phospho-specific antibodies, followed by incubation with a labeled secondary antibody and subsequent detection of the phosphorylated proteins [51].

Protein microarrays are miniaturized immunoassays that allow the identification of phosphorylated proteins through the detection of antibody–antigen interactions. Microarrays effectively analyze thousands of proteins without the need to pre-fractionate or enrich complex samples. The high multiplex of reverse phase protein microarrays (RPPAs) makes them powerful tools as they allow a side-by-side analysis of potentially thousands of proteins and phosphosites present within crude cellular or tissue lysates [52]. Furthermore, RPPAs require small amounts of a sample which is of huge benefit in clinical settings where limited amounts of samples are available [53]. The ability of RPPAs to analyze phosphorylated proteins and map cell signaling pathways has led researchers to examine their potential as a tool for the identification of patient-specific molecular patterns and, thus, patient-specific therapies [54,55]. However, as with all immunoassays, RPPAs are dependent on the availability of highly specific and sensitive antibodies. This is a limiting factor in the analysis of PTMs and the use of low-quality antibodies can lead to false positives and false negatives. Furthermore, interactions between proteins in a complex sample can also result in the masking of epitopes required for antibody binding [52]. Another antibody-based technique, immunohistochemistry, involves the use of phospho-specific antibodies to detect phosphorylated residues in tissue sections which can be visualized by staining. This relatively simple and cost-effective technique is widely used in research and clinical practice for diagnostic and prognostic purposes [56].

Phospho-specific flow cytometry or phospho-flow (to differentiate it from live-cell flow cytometry) demonstrates how FCM can be combined with PTM-specific antibodies for biomarker discovery and/or therapeutic target identification applications [57]. Incorporating phospho-flow techniques in the clinic to characterize phosphorylation events and signal transduction networks associated with disease at the cellular level is a promising approach in precision medicine. As more PTM-specific antibodies become available, the applications of FCM will increase with respect to detecting and quantitating distinctive modifications, with implications in diagnostic and predictive medicine.

MS-based techniques are powerful tools used in the identification and quantitation of phosphorylated proteins. Common methods of phosphopeptide enrichment include strong anion/cation exchange (SAX, SCX), hydrophilic interaction liquid chromatography (HILIC), immobilized metal ion affinity chromatography (IMAC), and metal oxide affinity chromatography (MOAC) [58,59,60,61,62]. Chemical methods such as β-elimination of the phosphoryl group on phosphorylated residues and the linkage of phospho-serine, -tyrosine and -threonine by chemical means, are less commonly used methods of phosphopeptide enrichment [63,64].

Ion exchange chromatography, including SAX, SCX and HILIC, is often used to reduce the complexity of biological samples. SCX is a relatively straightforward and efficient high performance liquid chromatography (HPLC) method used for the enrichment of phosphorylated and acetylated proteins [65]. Following tryptic digestion, most non-phosphorylated peptides have a net charge of +2 at a low pH due to the presence of a lysine or arginine residue at the C-terminal and a free amine group at the N-terminal. Concurrently, at a low pH, the hydroxyl group of phosphorylated residues is negative, resulting in phosphopeptides having a +1 charge. As the SCX column contains a negatively charged stationary phase, phosphorylated peptides (+1) will co-elute at an earlier stage than the unmodified tryptic peptides (+2) [58,65,66]. HILIC is a more powerful alternative to SCX, where separation is based on the hydrophilic nature of phosphorylated peptides. A polar stationary phase, such as TSK gel amide, and a less polar, organic mobile phase such as acetonitrile and water, are used during HILIC. The highly polar phosphopeptides interact with the polar stationary phase and are thus eluted at a later stage than unmodified peptides [48,67]. A variant of HILIC, termed electrostatic repulsion HILIC (ERLIC), utilizes a combination of hydrophilic interactions and electrostatic repulsion in order to separate phosphorylated and un-phosphorylated peptides [68].

Immobilized metal ion affinity chromatography (IMAC) exploits the affinity of phosphopeptides for positively charged metal ions such as Fe^3+^, Ga^3+^, and Zr^4+^. The chosen metal ions, commonly Fe^3+^, are non-covalently attached to a carrier resin. Sample peptides are incubated with the IMAC resin where the negatively charged phosphopeptides electrostatically interact with the positively charged resin [48,69]. A drawback of this enrichment technique is that non-phosphorylated peptides with many negatively charged residues can interact with the IMAC resin [70]. The conventional IMAC technique has been adapted and improved in recent years. Zhou et al. altered the IMAC technique by using a different resin containing Ti^4+^ cations [71]. This technique exceeded the performance of IMAC methods using Fe^3+^ and Zr^4+^ metal ions. Additionally, the application of dendrimers as IMAC matrices has shown promising results in various studies [72,73,74]. Zou, Jie and Yang [74] introduced a novel method of simultaneous phosphopeptide and N-glycosylation enrichment through the immobilization of Ti^4+^ cations onto a dendritic polyglycerol coated with chitosan, poly (glycidyl methacrylate) and iminodiacetic acid nanomaterials. The use of this highly sensitive and specific enrichment technique led to the identification of 423 N-glycopeptides and 422 phosphopeptides following LC–MS/MS [74]. A recent study demonstrated the improved performance of Zr-IMAC, Ti-IMAC, and TiO2-MOAC following optimization of the binding solvent used during the enrichment protocol. Following optimization, a comparison of the number of phosphopeptides identified between these techniques, as well as the most commonly used Fe-IMAC, revealed that microparticle-based Zr-IMAC enrichment was superior resulting in a higher phosphoproteome coverage [75]. This study also reinforced the widely documented view that combining enrichment techniques results in more robust PTM-focused MS-based analyses.

Metal oxide affinity chromatography (MOAC) is based on the affinity of the negatively charged phosphate group to metal oxides such as TiO_2_ or ZrO_2_. Phosphopeptides bind to the metal TiO_2_ stationary phase at a low pH and are subsequently separated from unmodified peptides during elution using an alkaline mobile phase [70,76,77]. During PTM analysis, fractionation and enrichment techniques are often combined to reduce sample complexity, enhance selectivity and increase the number of modified peptides identified [78,79,80]. For example, the combination of MOAC and IMAC, known as sequential elution from IMAC (SIMAC), allows for the capture of multi-phosphorylated peptides during the IMAC step followed by the capture of mono-phosphorylated peptides, from the unbound fraction of the first step, on the TiO_2_ column [81]. Furthermore, SIMAC has been combined with HILIC in an enrichment technique known as TiO_2_-SIMAC-HILIC (TiSH) to facilitate comprehensive phosphoproteome analysis with limited sample amounts [82].

Protein modifications have been reported to influence ionization efficiency during MS analysis. Using synthetically modified and unmodified peptides, Gropengiesser et al. [83] reported reduced ionization efficiencies of phosphorylated compared to unphosphorylated peptides during MALDI–MS and ESI–MS, with a greater reduction clearly present during MALDI–MS [83]. Although, the suggestion that phosphopeptides have lower ionization efficiencies is disputed by other researchers [84,85]. During phosphoproteomic MS-based analysis, CID fragmentation results in the cleavage of the labile phosphoester bond causing a neutral loss of H_3_PO_4_ (98 Da) from phosphorylated serine and threonine residues and the loss of HPO_3_ (80 Da) from phosphotyrosine residues, which appear as dominant peaks on MS spectra [86]. Extensive neutral loss can complicate phosphopeptide identification and site localization due to the distribution of numerous fragment ions across mass spectra. HCD, or beam-type CID, has been shown to produce less neutral loss peaks through higher-energy collisions [87]. ETD maintains the residue-bound phosphate group enabling conclusive phosphosite localization [69]. The detection of phosphorylation is based on the tryptic peptides demonstrating a mass shift of 80 Da and/or 98 Da, corresponding to the neutral loss of HPO_3_ and H_3_PO_4_, respectively [88]. The quantitation of phosphopeptides may employ label or label-free techniques. Labeling strategies used for phosphopeptide quantification include SILAC, iTRAQ and TMT, as described above [89].

### 2.2. Ubiquitination

Ubiquitination plays a key role in many cellular processes such as signal transduction, transcriptional regulation, DNA repair, intracellular localization and, most notably, the tagging of proteins for transfer to the proteasome for degradation. Abnormal ubiquitination can lead to enhanced or reduced degradation of specific proteins, hyperactive or inactive cell signaling pathways and an overall altered homeostasis in the cell [2]. A multienzyme cascade constituting ubiquitin-activating (E1), ubiquitin-conjugating (E2) and ubiquitin-ligating (E3) enzymes, results in the covalent attachment of the 76 amino acid protein, ubiquitin (Ub), to a lysine residue on the target protein. Ubiquitination is a highly transient modification with deubiquitination catalyzed by the deubiquitinating enzymes (DUBs) [90].

Within the last decade, promising efforts have been made to enhance current methods of protein ubiquitination analysis. A bottom-up proteomic approach incorporating trypsin is typically used resulting in the cleavage of ubiquitinated proteins after lysine and arginine residues. As Ub is covalently bound to a lysine residue, there is a missed cleavage at this residue followed by a cleavage after the first arginine residue of Ub, leaving two glycine (di-Gly) residues attached to the ubiquitinated peptide. Thus, during MS analysis of ubiquitinated peptides, there will be a mass shift of 114.043Da and a missed cleavage at the lysine residue which can be used to identify and localize ubiquitination sites [91]. The development of highly specific antibodies against the di-Gly remnant has greatly enhanced ubiquitinome research, facilitating immuno-based assays and the use of immunoaffinity purification and immunoprecipitation to enrich ubiquitinated proteins in complex samples prior to MS [92]. Kim et al. identified ~19,000 ubiquitination sites in ~5000 proteins using a di-Gly antibody to enrich for ubiquitinated proteins by immunoprecipitation [93]. The identification of ubiquitination sites can be determined through site-directed mutagenesis, where lysine residues are mutated into arginine, or in some cases cysteine. This inhibits ubiquitination of this residue and the sites are identified based on the exclusion principle. The occasional promiscuous nature of ubiquitin binding is a limiting factor of this method, leading to inaccurate localization of ubiquitination sites [94].

Once again, MS-based techniques are the most powerful tools used to analyze ubiquitination. As with phosphorylation, MS-based analysis of ubiquitination requires prior enrichment of ubiquitinated peptides. Both ESI and MALDI ionization techniques are used for ubiquitinome analysis [95,96]. Regarding fragmentation, ETD was reported to identify a higher number of ubiquitinated peptides when compared CID and HCD fragmentation methods [97]. The quantification and comparison of ubiquitinated peptide levels in different samples can be achieved using labelling technologies. SILAC is commonly used to analyze ubiquitination. However, refined labeling products such as iTRAQ and TMT have become more popular in recent years [18]. A recent study described a highly sensitive, rapid protocol, termed UbiFast, for deep analysis of the ubiquitinome using an on-antibody TMT labelling approach following di-Gly immunoaffinity enrichment of ubiquitinated peptides [98].

Determining whether a protein is mono-ubiquitinated or polyubiquitinated; and which lysine residue is utilized to form polyubiquitinated chains, is a crucial aspect when analyzing ubiquitination as these factors can indicate the functionality of the modification. For example, lysine 48 (K48) linkages target proteins for proteasomal degradation [90,99]. Ubiquitin-Absolute quantification (Ub-AQUA) is a quantitative technique capable of elucidating the specific linkages present on polyubiquitinated chains [100]. AQUA involves the generation of isotopically labeled standard peptides with ubiquitinated and un-ubiquitinated lysine residues. The standards and test sample co-elute during selected reaction monitoring (SRM), and the standards are used to quantify the linkages present within the sample [90,101]. Methods to analyze specific lysine linkages exploit the affinity of ubiquitin for ubiquitin-binding domains (UBDs) and tandem-repeated ubiquitin-binding entities (TUBEs) present on recombinant proteins. Wu et al. generated protein microarrays with bound recombinant UBDs to identify proteins that interact with K63-linked ubiquitin chains [102]. A recently described live-cell, high-throughput, luminescence-based assay incorporates the NanoBiT protein–protein interaction system and TUBEs to detect endogenous ubiquitination [103]. Tagged TUBEs interact with polyubiquitinated chains and can be separated from unmodified proteins and undergo further analysis of the specific linkages during MS [90,104]. Certain branched ubiquitin chains also display specific functions. For example, branched chains containing Lys-11 and Lys-48 linkages play a role in regulating the cell cycle through the degradation of key proteins, such as the cell cycle-regulated kinase, NEK2A [105]. The Ubiquitin Chain Enrichment Middle-down Mass Spectrometry (UbiChEM–MS) method incorporates a non-denaturing, limited trypsinolysis step to produce intact ubiquitin chains containing the di-Gly remnant of the branched chain at conjugated lysine residues allows the analysis of more than one ubiquitin chain present on a single ubiquitin molecule [106,107]. Wang and colleagues recently reviewed the current methods of branched ubiquitin chain detection [108].

### 2.3. Glycosylation

Glycosylation is the most common PTM, constituting almost half of all protein modifications. It plays a major role in the regulation of cellular processes such as protein structure and transport, cell metabolism and extracellular interactions. Glycosylation involves the attachment of sugar moieties, known as glycans, to target proteins in a process catalyzed by glycosyltransferases. There are various types of glycosylation, the most common being N-glycosylation followed by O-glycosylation. This PTM is one of the most complex PTMs due to the heterogeneity of glycan structures and their glycosylation sites [2,48].

Lectins are carbohydrate-binding proteins with specificities for certain glycan structures, often involved in the process of cell-to-cell interactions. The affinity of a various lectins for specific glycosylated proteins has been exploited and applied for the enrichment of N- and O-glycosylated proteins [109]. Antibodies have also been used in the analysis and enrichment of glycosylated proteins [110]; however, the highly dynamic nature of glycosylation causes difficulties during antibody-based methods. The enhanced stability and low-cost of lectins have made them the preferred tool in glycoprotein analysis [111]. Lectin affinity chromatography involves the separation of glycosylated proteins from non-glycosylated proteins in a complex sample [112]. Multi-lectin affinity columns incorporate lectins with specificities for certain glycoforms. These glycoforms are sequentially eluted, leading to fractions containing lectin-specific glycoproteins such as oligomannose, sialylation and fucosylation [113,114].

Chemistry-based methods have also been applied as enrichment strategies. Hydrazide chemistry is one of the most common methods for the enrichment of glycosylated proteins and involves the oxidation of glycans followed by their conjugation to a solid support through the formation of hydrazide bonds. The N-glycoproteins/peptides are cleaved and released using Peptide-N-Glycosidase F (PNGase F) [115,116]. During O-glycoprotein enrichment by hydrazide chemistry, O-glycoproteins/peptides can be released using hydroxylamine [117]. Boronic acid covalently interacts with *cis*-diol groups present on glycans and thus can be used to capture glycoproteins/peptides in a complex solution. After the non-glycosylated proteins have been washed through, the glycosylated proteins are released using an acidic solution [115]. HILIC, as described above, has also been applied for the enrichment of glycopeptides [118].

A promising enrichment method combining metabolic labeling and isotopic recording known as isotope targeted glycoproteomics (IsoTaG) has recently been developed [119]. The mass shift caused by the different isotopes allows the glycoproteins to be analyzed and sequenced by MS followed by the use of a specific software to identify glycoprotein structure and localization [115]. Another recently developed method combining chemical and enzymatic reactions known as solid phase extraction of N-linked glycans and glycosite-containing peptides (NGAG) facilitated the discovery of 2044 distinct N-glycosites [120].

Unlike phosphorylation or ubiquitination, the heterogeneity of glycans means there is no constant mass shift during MS analysis. Therefore, techniques to generate a common mass tag for MS analysis of glycoproteins have been developed. Enzymatic methods, such as the use of PNGase F and chemical methods, such as ammonium hydroxide/carbonate-based chemical deglycosylation have been applied to create a common mass tag for N-linked glycosylation [121,122]. β-elimination and the use of zinc finger nuclease (ZFN) to genetically alter cells and simplify the structure of glycans have been applied to create a common mass tag for O-linked glycosylation [123,124]. CID, ETD or HCD fragmentation methods may be used during MS analysis of glycosylation [48]. A combination approach for MS analysis of N-glycoproteins using HCD product ions to trigger ETD MS/MS has also been developed which facilitates targeted analysis of glycoproteins while eliminating the need for pre-enrichment of the complex sample [125].

### 2.4. Sumoylation

Sumoylation involves the covalent attachment of a small ubiquitin-like modifier (SUMO) to a lysine residue of target proteins through the formation of an isopeptide bond. In humans, there are three SUMO isoforms which are attached to proteins through an enzymatic cascade similar to the process of ubiquitination. The importance of sumoylation in many cellular processes including the cell cycle, transcriptional regulation, and nuclear localization has only recently been discovered [126,127,128]. The substoichiometric nature of sumoylation as well as the presence of high levels of active SUMO-specific proteases (SENPs) in cell lysates makes the analysis of this PTM challenging [129]. Furthermore, sumoylated peptides generated from tryptic digestion leave a large SUMO signature, >2 kDa or > 3 kDa depending on the isoform, resulting in complex MS spectra which hampers detection, sequencing, and identification of sumoylated peptides [130]. To overcome this challenge, various mutant SUMO tags have been created by mutation of specific amino acids into protease-cleavable residues to generate short peptide signatures. Examples of mutant SUMO tags include SUMO-1^T95R^, SUMO-2^T91R^ and SUMO-3^Q87R/Q88N^ [131,132]. The use of mutant SUMO tags greatly enhances MS resolution, leading to more sumoylation sites being identified. However, the identifications made using these ectopically expressed tags does not equivalate to endogenous sumoylation patterns and mutant SUMO tags are not compatible with the analysis of clinical samples [130]. To enrich for sumoylated peptides, an affinity tag, typically Histidine (His), is often conjugated to SUMO for purification by IMAC [133,134]. Site-directed mutagenesis of a predicted lysine acceptor residue is often used in small-scale studies to detect sumoylation [135,136].

Other non-mutant methods have been developed to detect endogenous sumoylation. These techniques often involve enriching sumoylated peptides by co-immunoprecipitation using anti-SUMO antibodies followed by protein digestion using various proteases such as trypsin, Lys-C, Asp-*N* and wild-type α-lytic protease (WaLP) [137,138,139]. A method derived to avoid the limitations associated with mutant SUMO peptides, protease-reliant identification of SUMO modification (PRISM), involves the use of His-tagged SUMO, acetylation and SUMO-specific proteases for sumoylation site identification [140]. Furthermore, Hendriks et al. identified 14,869 SUMO2/3 sites in human cells by incorporating a serial digestion workflow using Lys-C and Asp-*N* and peptide-level immunoprecipitation followed by LC–MS/MS [139].

SUMO-interacting motifs (SIMs) and recombinant SUMO-binding entities (SUBEs) have been adopted for the enrichment and identification of endogenous poly-SUMO proteins [141,142]. SILAC, iTRAQ and LFQ have been used for quantitation of sumoylation [138,143,144]. Various bioinformatic tools such as SUMmOn, SUMOhydro, SumSec, etc., have been introduced for sumoylation site identification [129,145,146,147].

### 2.5. Acetylation and Methylation

Acetylation and methylation are prominent PTMs that play roles in many cellular processes including cell signaling, metabolic pathways and most notably, DNA-protein interactions. The acetylation of histone proteins is a crucial process that influences the accessibility of DNA to the transcriptional machinery. The transfer of an acetyl group to the α-amino group at the N-terminus of the protein is an irreversible modification, whereas acetylation at a lysine residue is reversible. Acetylation is catalyzed by acetyltransferases and lysine acetylation may be reversed by lysine deacetylases [148]. Methylation mainly occurs on lysine and arginine amino acids. However, other residues such as histidine, proline, and glutamine may also be subject to methylation. Methylation is catalyzed by lysine or arginine methyltransferases and reversed by demethylases [149] Research on acetylation and methylation is often focused on histone modifications which requires specific sample preparation approaches, as described above [22].

Radiolabeling of proteins with radiolabeled acetyl groups from ^14^C or ^3^H-acetyl CoA allows the detection of acetylated proteins by autoradiography after gel electrophoresis is performed [150,151]. However, the use of this technique has dropped in recent years due to the extra safety precautions required when using radioactive molecules [148]. Immuno-based techniques are commonly used to detect acetylation with many highly specific and sensitive anti-acetyl lysine antibodies currently available [152]. Several recent studies have expanded on conventional FCM for acetylation analysis using FCM-based techniques including single-cell imaging flow cytometry and mass cytometry (CyTOF) [153,154,155].

Once again, acetylated proteins are enriched prior to LC–MS/MS. Immunoaffinity purification of acetylated peptides using specific antibodies is the most common and effective method of enrichment [22,156,157,158]. Combined fractional diagonal chromatography (COFRADIC) is also a popular technique that specifically enriches N-terminal acetylated proteins through the derivatization of primary amines [159,160]. The relatively new method, stable-isotope protein N-terminal acetylation quantification (SILProNAQ) allows the direct quantitation of N-terminal peptides, although few studies have reported using this technique [161,162]. The previously discussed fractionation technique, SCX is commonly used for acetylation analysis. Tandem MS identifies acetylation by the presence of a 42 Da mass shift on acetylated peptides. The quantitation of acetylated proteins may be carried out using label or label-free quantification. SILAC, iTRAQ and TMT labeling have been applied for the quantification of acetylated proteins [163,164,165].

Typically, methylated proteins are detected by LC–MS/MS. However, the development of effective enrichment strategies and the accurate localization of methylation sites has lacked the same success as other PTMs such as phosphorylation, due to the small size of the methyl group, difficulties assigning the mass shifts for mono-, di- and tri-methylation (14 Da, 28 Da and 42 Da, respectively), and the lack of significant physicochemical changes between methylated and unmethylated lysine and arginine residues [166,167,168]. The previously described enrichment methods HILIC and SCX as well as isoelectric focusing (IEF) have been applied. However, the overall consensus suggests that orthogonal combination of immunoaffinity enrichment and ion exchange chromatography techniques enhances the robustness of methylome analysis [169,170,171]. Specific antibodies have been developed against mono- and di-methylated arginine as well as mono-, di- and tri-methylated lysine, leading to the identification and localization of over 1000 arginine methylation sites and approximately 160 lysine methylation sites in the human HCT116 cell line [172]. Similar to TUBEs for ubiquitination analysis, methyl-binding domains have been exploited for enrichment purposes. The affinity of naturally occurring triple malignant tumor-binding domains (3×MBT) from the protein L3MBTL1, for mono- and di-methylated lysine residues of proteins resulted in the use of these domains for enrichment [173,174]. Recently, Wang et al. [175] described a new chromatography-based method of methylome analysis combining SCX, IMAC and high-pH reversed-phase chromatography which led to the identification of 765 methylation sites [175]. Positive- and negative-mode CID, HCD and ETD fragmentation techniques have been applied for methylation analysis [176]. For quantitation, label-based techniques such as heavy-methyl SILAC and isomethionine methyl-SILAC; or label-free techniques are often used [171,177,178,179].

**Figure 3:**
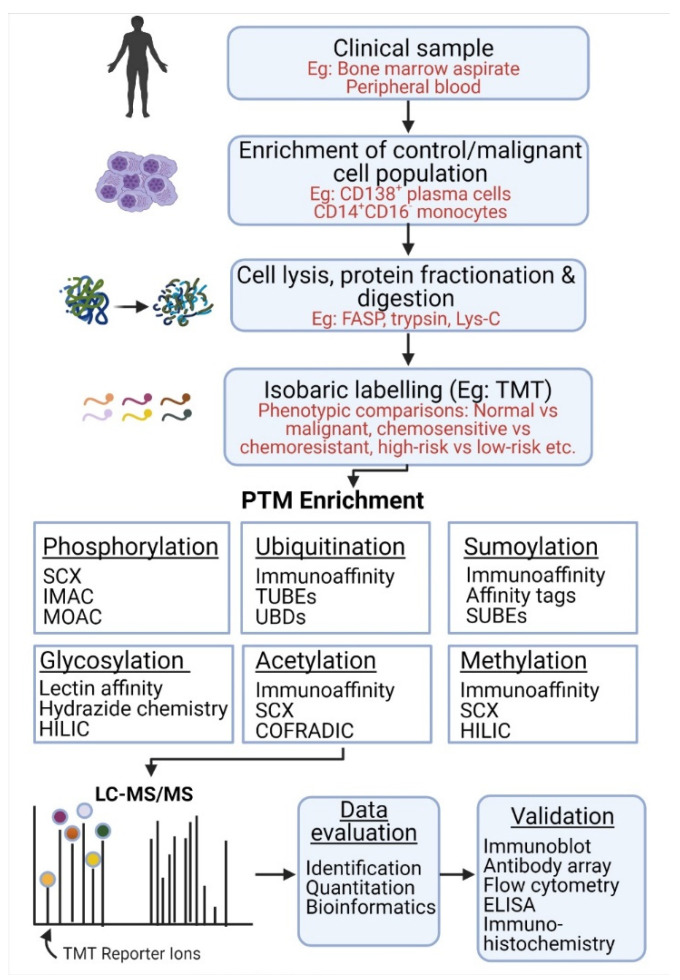
Bottom-up proteomics workflow for PTM analysis and subsequent validation. Bottom-up proteomic analyses are often applied for PTM-focused studies. This workflow can be adapted based on the PTM of interest through the selection of PTM-specific enrichment techniques. The examples provided in the figure (red font) highlight potential adaptations of this approach for blood cancer research. Middle-down and top-down proteomic approaches for PTM analysis are gaining popularity and may also present as useful techniques for future studies in blood cancer research [180,181]. FASP, filter-aided sample preparation; TMT, tandem mass tag; SCX, strong cation exchange chromatography; IMAC, immobilized metal ion affinity chromatography; MOAC, metal oxide affinity chromatography; TUBEs, tandem ubiquitin-binding entities, UBDs, ubiquitin-binding domains; SUBEs, SUMO-binding entities; HILIC, hydrophilic interaction liquid chromatography; COFRADIC, combined fractional diagonal chromatography. Created using BioRender.com.

The techniques described above can be incorporated into various experimental designs to identify and quantify PTMs associated with specific cellular processes and diseases. The bottom-up approach is among the most popular proteomic workflows which can be adapted for the study of specific biological samples and PTMs of interest (Figure 3).

## 3. PTM Crosstalk

As described, many methods have been developed to analyze individual PTMs. However, multiple PTMs on a target protein can positively or negatively affect the influence of each PTM on the target protein. This crosstalk between PTMs participates in the regulation of many molecular processes [182]. For example, ubiquitination, acetylation, and phosphorylation are involved in the regulation of the tumor-suppressor protein, p53 [183]. To analyze the interplay between PTMs, orthogonal combinations of enrichment and/or fractionation techniques for individual PTMs have been applied. The serial enrichment of different PTMs (SEPTM) method, developed by Mertins et al., [184] involves the sequential enrichment of phosphorylated, ubiquitinated and acetylated proteins before MS analysis. This method typically incorporates IMAC or TiO_2_ enrichment for phosphorylated proteins, immunoaffinity enrichment using di-Gly antibodies for ubiquitinated proteins and anti-acetyl lysine antibodies for acetylated proteins [184]. Further discussion on PTM crosstalk is beyond the scope of this review. However, many informative reviews can be found on this increasingly important topic [182,185].

## 4. Application of PTM-Focused Techniques in Blood Cancer Research

**Figure 4:**
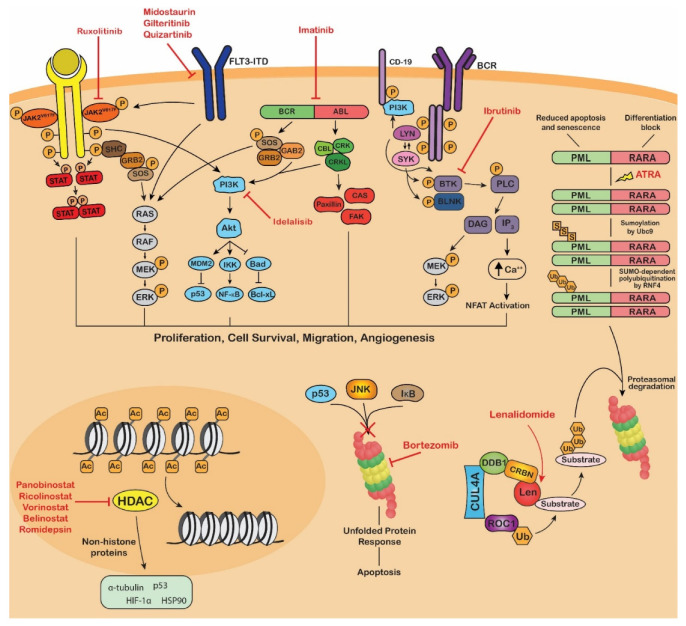
Targeting of protein modifications by clinically used blood cancer therapeutics. Ruxolitinib, midostaurin, gilteritinib, imatinib, dasatinib, nilotinib, bosutinib, ponatinib, ibrutinib and idelalisib are kinase inhibitors that inhibit the phosphorylation and activation of key proteins of oncogenic signaling pathways, promoting growth arrest and apoptosis in cancer cells. ATRA therapy for the treatment of APL induces SUMOylation-dependent polyubiquitination and proteasomal degradation of the fusion oncoprotein PML-RARA. The immunomodulatory drugs, lenalidomide, thalidomide and pomalidomide bind Cereblon, the substrate adaptor of the CRL4^CRBN^ E3 ubiquitin ligase, modulating its substrate specificity. Bortezomib, carfilzomib and ixazomib target the 26S proteasome, blocking the degradation of polyubiquitinated regulatory proteins and inducing the unfolded protein response resulting in apoptosis. Panobinostat, vorinostat, belinostat and romidepsin are HDAC inhibitors that block the deacetylation activity of HDACs and exert their anti-tumor activity through modulating the acetylation status of a variety of histone and non-histone proteins. JAK2^V617F^, Janus kinase 2 V617F; STAT, signal transducer and activator of transcription; SHC, Src homology and Collagen; GRB2, growth factor receptor-bound protein 2; SOS, Son of sevenless; RAF, RAF proto-oncogene serine/threonine-protein kinase; MEK, Mitogen-activated protein kinase kinase; ERK, extracellular signal-regulated kinase; FLT3-ITD, fms like tyrosine kinase 3 – internal tandem duplications; PI3K, phosphatidylinositol 3-kinase; MDM2, mouse double minute 2 homolog; IKK, IκB kinase; Bad, BCL2 associated agonist of cell death; NF-κB, Nuclear factor kappa B; Bcl-xL, B-cell lymphoma-extra large; GAB2, GRB2 associated binding protein 2; BCR-ABL, breakpoint cluster region-proto-oncogene tyrosine-protein kinase; CBL, E3 ubiquitin-protein ligase CBL; CRK, Proto-oncogene c-Crk; CRKL, Crk-like protein; CAS, Crk-associated substrate; FAK, focal adhesion kinase; BCR, B-cell receptor; Lyn, tyrosine-protein kinase Lyn; SYK, tyrosine-protein kinase SYK; BTK, Bruton’s tyrosine kinase; BLNK, B-cell linker protein; PLC, phospholipase C; DAG, diacyl glycerol; IP_3_, inositol 1,4,5-trisphosphate; NFAT, nuclear factor of activated T-cells; PML-RARA, promyelocytic leukemia/retinoic acid receptor alpha; ATRA, all-trans retinoic acid; Ubc9, SUMO-conjugating enzyme UBC9; RNF4, ring finger protein 4; CUL4A, cullin 4A; DDB1, DNA damage binding protein 1; CRBN, cereblon; ROC1, regulator of cullins-1; Len, lenalidomide; JNK, c-Jun N-terminal kinase (JNK); IκB, inhibitor of nuclear factor kappa B; HIF-1α, hypoxia-inducible factor 1-alpha; HSP90, heat shock protein 90; P, phosphorylation; S, sumoylation; Ub, ubiquitination; Ac, acetylation.

Several reviews have been published focusing on specific PTMs and their involvement in carcinogenesis. However, literature surrounding the relationship between PTMs and hematological malignancies is limited [186,187,188]. Both the individual PTMs and associated modifying enzymes play significant roles as biomarkers and therapeutic targets in hematological malignancies [189]. Many signaling pathways regulated by PTMs have demonstrated aberrant activity in blood cancers, such as the Janus Kinase (JAK)/signal transducer and activator of transcription (STAT), phosphatidylinositol-3-kinase (PI3K)/AKT and B-cell receptor (BCR) signaling pathways (Figure 4). Dysregulation of these pathways cause proliferation, migration, survival, and angiogenesis which promote and/or maintain the malignant phenotype of malignant blood cells. Several genetic abnormalities associated with hematological malignancies have also been found to affect protein modifier genes, such as the t(9;22) translocation which results in the oncogenic BCR-ABL fusion kinase (Figure 4) [190]. Already, many FDA-approved therapies for hematological malignancies incorporate the modulation or inhibition of oncogenic protein modifications as part of their mode of action (Table 1, Figure 4). Therefore, the application of efficient analytical techniques in laboratories globally for thorough PTM analysis may aid in the discovery of novel biomarkers and therapeutic targets in hematological malignancies.

MS-based techniques are typically used for large-scale systematic analysis of PTM marks in blood cancers, generating extensive data. Bioinformatic analysis of this data can identify significant changes in the patterns of protein modification between disease states, such as malignant versus normal samples. Further research can then be conducted to determine the importance and implications of specific PTM changes in blood cancers. The technique of choice for validation and small-scale analyses is Western blot analysis using antibodies specific to the PTM being analyzed.

## 5. Multiple Myeloma

Multiple myeloma (MM) is an incurable hematological malignancy characterized by the uncontrolled proliferation of monoclonal plasma cells in the bone marrow resulting in end-organ damage, renal injury, and the destruction of bone tissue [213]. Despite being considered incurable, the survival of MM patients has dramatically improved in recent decades with treatment consisting of therapeutic agents including corticosteroids, immunomodulatory agents (IMiDs), proteasome inhibitors and monoclonal antibodies (MAbs) [214]. The analysis of PTMs in MM has identified potential biomarkers and therapeutic targets with the application of the techniques described above being central to these findings (Table 2).

PTM analysis in MM has revealed aberrant modifications of major proteins involved in signaling pathways known to play a role in MM carcinogenesis and progression, such as JAK/STAT signaling, PI3K/Akt/mammalian target of rapamycin (mTOR) and Ras/Raf/MEK/Erk. Targeting kinases, phosphatases, E3 ubiquitin ligases, deubiquitinating enzymes (DUBs) and histone deacetylases (HDACs) showing altered activity resulting in aberrant protein modifications in these signaling pathways has demonstrated promising results in the form of identifying novel therapeutic targets to reduce MM growth and targeting resistance mechanisms to current MM therapies, such as bortezomib (Table 2). A large-scale MS/MS-based phosphoproteomic analysis, incorporating TiO_2_ phosphopeptide enrichment, on a primary MM cell line by Ge et al. identified many novel phosphosites and provided a basis for future phosphorylation-based research in MM [231]. A more recent large-scale MS-based study utilizing phosphoproteomic techniques, such as IMAC phosphopeptide enrichment, and transcriptomic techniques revealed dynamic kinase activity and differential phosphorylation signatures in *KRAS-* and *NRAS*-mutated MM. This study supports the need for improved patient stratification, indicating the potential benefit of incorporating the level of kinase activity as well as the mutational status of the patient during therapeutic decision making [217]. Western blot analysis has identified altered phosphorylation of several important regulatory proteins including EZH2, AMPK, p53, STAT3, mTOR and ERK1/2, which promote MM growth and survival, thus identifying several potential therapeutic targets and enhancing our understanding of MM pathogenesis (Table 2). Brown et al. conducted a phospho flow study to detect constitutive and IL-6-induced phosphorylation of key signaling molecules in MM, demonstrating the potential of this technique to detect novel diagnostic and prognostic phosphoproteomic biomarkers [232].

The introduction of proteasome inhibitors, such as bortezomib, revolutionized the treatment of MM and patient survival. Bortezomib exerts its anti-myeloma activity by blocking the proteasomal degradation of ubiquitinated tumor suppressing regulatory proteins, such as p53. Due to the development of drug resistance, targeting other components of the ubiquitin-proteasome system has become an attractive concept. A recent preclinical study illustrated the potential of an E1 ubiquitin activating enzyme (UAE) inhibitor, TAK-243, for the treatment of MM [233]. Approved IMiDs target the ubiquitin proteasome system by modulating the substrate specificity E3 ubiquitin ligases to exert their anti-cancer effects. Lenalidomide targets Cereblon, the substrate recognition subunit of a biologically important Cullin RING E3 ubiquitin ligase complex, resulting in the degradation of two B-cell transcription factors, Ikaros (IKZF1) and Aiolos (IKZF3), that are essential for the survival of MM cells [207]. Inhibition of the DUB, USP1, has also been shown to induce apoptosis in myeloma cells [234]. A recent review by Wirth et al. provides an in-depth discussion on ubiquitination and ubiquitin-like modifications in MM [235].

Altered methylation and acetylation of histones and non-histones have been implicated in MM. Deacetylation of c-MYC and DNMT1 by HDAC3 was found to stabilize these oncogenic proteins and promote MM cell survival [227]. MMSET and EZH2 have histone methyltransferase activity and are associated with MM progression and a poor prognosis [236,237]. Panobinostat is an FDA-approved pan-HDAC inhibitor used in the treatment of relapsed or refractory MM [238]. HDAC inhibitors have been found to improve the efficacy of treatment with proteasome inhibitors by blocking the activation of an alternative non-proteasomal degradation pathway, the aggresome/autophagy pathway via the inhibition of HDAC6-mediated transport of polyubiquitinated proteins to aggresomes for degradation [239,240,241]. Other HDAC inhibitors, ricolinostat and citarinostat, are currently in clinical trials for the treatment of MM [240,242].

The dysregulation of the SUMOylation pathway in MM described by Driscoll et al. [224] has led to further research into the targeting of SUMOylation machinery. Upregulation of the SUMO-conjugating enzyme UBE21 and the SUMO E3 ligase PIAS1 are markers of poor prognosis in MM [224]. Aberrant expression of SENPs has been implicated in the constitutive activation of the oncogenic NF-κB pathway in MM. The overexpression of SENP1 causes enhanced proliferation, whereas the silencing of SENP2 induces bortezomib resistance in MM cells [223,243]. Glycosylation analysis incorporating lectin histochemistry demonstrated that inhibiting sialyation prevents MM cells from entering the bone marrow microenvironment, in which they are often protected from conventional chemotherapy; presenting sialyation as a promising target to improve current MM therapies [226]. As can be seen in Table 1, through the application of PTM-focused analytical techniques, significant progress has been made in elucidating factors involved in MM pathogenesis and their potential as therapeutic targets and biomarkers.

## 6. Acute Myeloid Leukemia

Acute myeloid leukemia (AML) is a highly heterogeneous disease of the blood and bone marrow characterized by the uncontrolled proliferation and altered differentiation of myelogenous progenitor or stem cells. The complexity of AML is illustrated by its classification into six disease subtypes based on distinct differences in genetic make-up, morphology and clinical presentation of AML [244]. Progress in understanding the pathobiology of AML has led to the FDA approval of eight therapies since 2017 [245]. PTM analysis has improved the understanding of altered signaling in AML and identified several PTM marks as potential therapeutic targets (Table 3). Interfering with protein modification in AML using tyrosine kinase inhibitors, hypomethylating agents (HMAs), histone deacetylase (HDAC) inhibitors, as well as ubiquitination and SUMOylation inhibitors, has led to considerable progress in the improvement of treatment for AML.

Mutations in the gene encoding the receptor kinase, FMS-like tyrosine kinase 3 (FLT3), are poor prognostic markers found in over 30% of AML cases [245]. Proteomic analysis has revealed that upon ligand binding, FLT3 initiates a phosphorylation cascade resulting in the activation of proliferative signaling pathways [261]. FLT3-internal tandem duplication (ITD) mutants exert pro-proliferative effects through the constitutive activation of STAT5 signaling [262]. Under normal circumstances, FLT3 undergoes glycosylation in the endoplasmic reticulum (ER) and Golgi apparatus to form a mature 160 kDa protein that localizes at the cell surface. In FLT3-ITD^+^ AML, there is partial retention of the underglycosylated form of FLT3-ITD in the ER. This 130 kDa autophosphorylated isoform stimulates STAT5 signaling, upregulating the oncogenic kinase PIM-1 resulting in the establishment of a positive feedback loop promoting constitutive STAT5 signaling [257]. Targeting of this phosphorylation cascade has led to the approval of three FLT3 tyrosine kinase inhibitors (TKIs) in recent years for the treatment of AML with FLT3 mutations (Figure 4) [263]. Phase 1 studies of the pan-PIM kinase inhibitor, AZD1208, in patients with relapsed or refractory AML revealed no clinical response when administered as a single agent [264]. However, a recent study found that AZD1208 in combination with the proteasome inhibitor, bortezomib, enhanced bortezomib-induced apoptosis [265]. The inhibition of N-linked glycosylation in AML cells using, 2-deoxy-d-glucose (2-DG), reduced tumor growth. However, further studies are required to translate these results into a competent therapy [266].

Unlike other AML subtypes, curative treatment of acute promyelocytic leukemia (APL) has been hugely successful using a combination of all-trans-retinoic acid (ATRA) and arsenic trioxide (ATO) to restore the ability of leukemic cells to differentiate. The mechanism of action of this treatment involves the SUMOylation, ubiquitination and subsequent proteasomal degradation of the PML/RARα fusion oncoprotein [267]. Recent studies have shown that the ubiquitination and degradation of the cyclin-dependent kinase 2 (CDK2) causes differentiation of AML cells, thus demonstrating the potential of CDK2 inhibitors as differentiation agents in AML [251]. The UAE inhibitor, TAK-243, has demonstrated anti-leukemic activity in preclinical studies, with a phase 1 clinical trial for the treatment of relapsed or refractory AML currently ongoing [253]. Studies have also identified the E3 ubiquitin ligases, TRIAD1 and Cbl-b, as tumor suppressors in AML opening avenues for further investigation of their downstream substrates as potential therapeutic targets [127,252]. Western blot analysis and site-directed mutagenesis of specific lysine residues determined that the sumoylation of the transcription factor, sPRDM16, and insulin-like growth factor 1 receptor (IGF-1R) promote leukemogenesis [254,268].

Analysis of the acetylome and methylome using a combination of MS-based and antibody-based techniques has provided insights into aberrant epigenetic regulation in AML. Compounds have been developed to target commonly overexpressed or hyperactive HDACs, histone methyltransferases and demethylases, with several clinical trials evaluating their efficacy against AML in progress [269,270]. These epigenetic therapies show low levels of toxicity and have proved to be promising agents for the treatment of aging AML where patients are unable to tolerate intensive chemotherapy [269]. Taken together, investigating the role of altered protein modifications on the development and progression of leukemogenesis has contributed to the discovery of novel biomarkers and therapeutics targets in AML.

## 7. Myeloproliferative Neoplasms

Myeloproliferative neoplasms (MPNs) are a group of disorders derived from hematopoietic and progenitor stem cells (HPSCs). They are associated with enhanced proliferation of myelogenous cells, with specific MPNs often characterized by genetic mutations. Chronic myeloid leukemia (CML) is characterized by the *BCR–ABL1* fusion gene mutation whereas polycythemia vera (PV), essential thrombocythemia (ET) and myelofibrosis (MF) are typically characterized by the presence of *JAK2, CALR*, or *MPL* gene mutations [271]. These neoplasms develop slowly but have an increased risk of disease progression to the more severe myeloproliferative neoplasm, primary myelofibrosis (PMF), or AML [272]. Myelodysplastic syndromes (MDS) are clonal hematopoietic disorders often characterized by ineffective hematopoiesis, a dysplastic morphology of one or more myelogenous cell types and peripheral cytopenia [273]. Similar to MPNs, these disorders are associated with genetic alterations in a variety of genes such as *TET2* and *ASXL1*, and an increased risk of transformation to AML [274,275]. Few large-scale PTM analyses have been carried out on MPNs and MDS compared to AML and MM. Research is often focused on genomic analysis due to the contribution of genomic abnormalities and somatic mutations to disease progression [275,276]. Despite this, MS-based analysis, protein microarrays and, most commonly, Western blot analysis have been applied in various studies, aiding in the identification of dysregulated modifications and potential therapeutic targets in MPNs and MDS (Table 4).

The JAK2^V617F^ mutation, found in a high proportion of patients with MPNs, results in the phosphorylation of JAK2 in the absence of cytokine stimulation, leading to the constitutive phosphorylation and activation of the downstream target proteins, STAT and ERK, and the development of a pro-proliferative phenotype [272]. The efficacy of the FDA-approved JAK1 and JAK2 inhibitor, ruxolitinib, for the treatment of JAK2^V617F^-MPNs, mainly MF and PV, has been varied [291]. In comparison, the inhibition of the kinase activity of BCR-ABL in CML by the FDA-approved imatinib, nilotinib, dasatinib, bosutinib and ponatinib, has demonstrated huge success with an increase in complete cytogenetic responses and improved overall survival [292]. Phospho-analysis has identified the upregulation of the programmed death ligand 1 (PD-L1) and programmed death receptor 1 (PD-1) in JAK2^V617F^-mutant MPN, as well as increased RalA which promotes disease progression in CML [278,280]. A phase I-II clinical trial (ClinicalTrials.gov Identifier: NCT04051307) in PV and ET patients investigating the safety and efficacy of vaccinating with PD-L1 and Aginase1 peptides is currently in the recruitment stage.

The Asrij/OCIAD1 protein prevents MDM2-mediated ubiquitination and degradation of p53, thus promoting hematopoietic stem cell quiescence. The modulation of p53 ubiquitination by Asrii could be used as a base for further research into targeted therapies for MPNs with dysregulated p53 [281]. Using a combination of genomic, transcriptomic and antibody-based proteomic techniques, an MDS-associated germline mutation in the *HLTF* gene was found to impair polyubiquitination of proliferating cell nuclear antigen (PCNA) causing decreased DNA repair and an accumulation of DNA damage [293]. Limited studies have been conducted on the involvement of SUMOylation in MPNs. One study revealed that the β-catenin antagonist, Chibby 1 (CBY1), is downmodulated in CML due to enhanced SUMOylation, leading to the aberrant activation of β-catenin. This provides an insight into the potential role of SUMOylation in pathogenic molecular pathways contributing to MPNs. Furthermore, the inhibition of the E3 ligase responsible for the enhanced SUMOyation of CBY1 may be a promising avenue for further research [285]. Impaired glycosylation of the colony stimulating factor 3 receptor (CSF3R) and the thrombopoietin receptor (TpoR) resulting in the constituent activation of the JAK/STAT pathway has been identified in MPNs [286,287]. ASXL1, a commonly mutated gene in MDS, was recently found to be part of a complex with O-GlcNAc transferase (OGT) in which OGT stabilizes ASXL1 via O-glycosylation. Disruption of this complex blocks myeloid differentiation via the inhibition of H3K4 methylation, providing insight into a possible mechanism of MDS pathogenesis [294]. HDAC inhibitors and hypomethylating agents are promising therapeutics that have demonstrated efficacy in MPNs when combined with ruxolitinib [295,296]. Mutant JAK2 phosphorylates the arginine methyltransferase, PRMT5, impairing its ability to methylate target proteins resulting in a myeloproliferative phenotype in JAK2^V617F^-mutant patient samples [297]. Inhibitors of class I and II HDACs, givinostat, panobinostat and vorinostat, have demonstrated clinical efficacy in various MPNs [295]. A recent study demonstrated that the class IV HDAC, HDAC11, rather than HDAC6 stimulates proliferation and oncogenic hematopoiesis in JAK2-driven MPNs and is a promising therapeutic target for future research [289]. A quantitative MS-based analysis identified differentially expressed histone marks as candidate markers of MDS progression in an early-stage MDS and late-stage leukemic cell line, as well as histone marks with potential relevance in resistance to the standard of care hypomethylating agent, decitabine [298]. PTM analysis has identified various potential biomarkers and therapeutic targets in MPNs and MDS as well as specific proteins involved in the modification of proteins, such as JAK2, which are targets of current FDA-approved therapies.

## 8. Lymphomas

Lymphomas are a heterogenous group of almost 100 distinct B- and T-cell malignancies that develop from the oncogenic transformation of normal lymphoid cells [299]. The majority of PTM-based lymphoma research focuses on phosphorylation, with a limited number of studies focusing on other PTMs (Table 5). Studies incorporating phospho-analysis have identified changes in protein phosphorylation levels in normal compared to lymphoma cells; mechanisms of lymphomagenesis and tumor survival; and modes of action of potential therapies [300,301,302]. Large-scale quantitative phosphoproteomic analysis has given insights into the signaling pathways involved in the development and progression of lymphomas. MS-based analysis of chimeric antigen receptor (CAR)- stimulated phosphorylation events utilizing an IMAC enrichment technique and TMT labeling identified 26,804 unique phosphorylation sites [303]. CAR T cell therapy is FDA approved for the treatment of several large B cell lymphomas [304]. This study compared phosphorylation-mediated signaling pathways in CD28/CD3ζ and 4–1BB/CD3ζ CAR T cells, providing important information for future improvements in CAR design to enhance clinical efficacy [303]. A semiquantitative, label-free, MS-based analysis of B-cell non-Hodgkin lymphomas (NHL) mapped phosphorylation sites and created distinct phosphorylation signatures in different NHLs [305]. A recent focused study on anaplastic large-cell lymphomas (ALCLs) expressing the oncogenic fusion kinase NPM-ALK, identified NPM-ALK targeted phosphorylation sites on nuclear interaction partner of ALK (NIPA) which promote lymphomagenesis when phosphorylated [306]. Dysregulation of B cell receptor (BCR) signaling, through constitutive phosphorylation of downstream targets, contributes to the growth and survival of many B cell lymphomas. TKIs, such as ibrutinib and idelalisib, target kinases involved in BCR signaling [307].

Viral infections can drive lymphomagenesis and disease progression through the activation of signaling pathways by viral oncogenic proteins. Activation of PIK3/AKT signaling and STAT3 via constitutive phosphorylation contribute to the growth and survival of primary effusion lymphoma (PEL) cells, a type of NHL driven by dual infection with Epstein Barr virus (EPV) and Kaposi sarcoma–associated herpesvirus (KSHV) [317,318]. Inhibition of STAT3 and PI3K signaling promotes apoptosis in PEL cells and dual inhibition of PI3K and mTOR kinases inhibits PEL cell proliferation, illustrating STAT3, PI3K and mTOR signaling as potential targets for the treatment of PEL [319]. Subsequently, a study by Mohanty et al. [320] revealed everolimus as a multi-targeted therapy that induces apoptosis and downregulates mTOR, STAT-3 and NF-κB signaling in KSHV+ PEL [320]. Recent kinome profiling of PEL cell lines using kinase inhibitor-conjugated beads to capture kinases followed by quantitative MS revealed Tyro3 as a potential therapeutic target with subsequent analyses revealing that Tyro3 inhibition reduced PEL survival and growth in vitro, and tumor burden in a mouse PEL xenograft model in vivo [321]. KSHV latency-associated nuclear antigen (LANA) was found to prevent degradation of the oncogene c-Myc in PEL cell lines by inhibiting glycogen synthase kinase-3β (GSK-3β)-mediated phosphorylation of the T58 residue of c-Myc [322].

Certain E2 ubiquitin-conjugating enzymes, E3 ubiquitin ligases and DUBs have been suggested to play a role in aberrant BCR and NF-κB signaling in lymphomas and represent potential targets for novel therapies [323]. Components of E3 ligases involved in lymphomagenesis include members of the Inhibitor of APoptosis (IAP) protein family, FBXO10, FBXO11, cereblon and TRIM11. Compounds targeting IAPs, known as Smac mimetics, are being investigated for the treatment of activated B cell-like (ABC) subtype of diffuse large B cell lymphoma (DLBCL) and rituximab-resistant B-cell lymphoma [309,324]. A recent study utilizing Ub-specific antibodies and ubiquitination assays, identified that upon BCR stimulation, FBXO10 acts as the critical component of a negative autoregulatory feedback loop via the ubiquitination and degradation of human germinal center-associated lymphoma (HGAL) protein [325]. Low FBXO10 expression levels and loss-of-function mutations in the FBXO10 gene have been identified in mantle cell lymphoma and DLBCL, respectively [325,326]. Cereblon is a direct target of IMiDs used in the treatment of several types of lymphoma [327]. SILAC-based quantitative MS analysis demonstrated that bromodomain and extraterminal protein family inhibitors downmodulate the E2-conjugating enzymes, UBE2C and UBE2T, promoting the anti-lymphoma activity of PI3K inhibitors [328]. DUBs, ubiquitin carboxyl-terminal hydrolase L5 (UCHL5) and the ubiquitin-specific peptidase 14 (USP14), were detected in the cytoplasm of a high proportion of DLBCL tumor cells [329]. UCHL5 and USP14 inhibitor, b-AP15, induces apoptosis in DLBCL cell lines and mouse xenograft models demonstrating a potential novel mechanism for DLBCL treatment [330]. Human T-cell leukemia virus type 1 (HTLV-1), the etiologic agent of adult T-cell leukemia/lymphoma, encodes several oncogenic proteins that promote ATLL progression, including Tax, a protein whose function and localization is largely regulated by PTMs [331]. A recent study provided evidence that UBE4B, an E3/E4 ubiquitin conjugation factor, is a Tax-binding protein that conjugates Tax with K48- and K63-linked polyubiquitin chains resulting in the downstream activation of NF- κB signaling in Tax^+^ ATLL [332].

A study, incorporating transcriptomics, immunoblotting and immunohistochemistry (SUMO-1,2,3 antibodies), showed an upregulation of components of the SUMOylation machinery in various Myc-overexpressing lymphomas. Furthermore, the inhibition of SUMOylation in Myc-driven lymphomas resulted in the induction of cell cycle arrest and apoptosis [333]. EBV-associated lymphomas have also shown dysregulated SUMOylation through the EBV latent membrane protein-1 (LMP1)-mediated interactions with SENP2 and Ubc9 [312,334]. Altered SUMOylation presents an avenue for possible therapeutic targeting of the SUMOylation pathway in lymphomas. A small-molecule inhibitor of SUMOylation, TAK-981, is currently being evaluated in clinical trials; in a phase I study in patients with metastatic solid tumors and lymphomas (ClinicalTrials.gov Identifier: NCT03648372) and in a phase Ib/II study in combination with rituximab in patients with relapsed or refractory CD20+ non-Hodgkins lymphoma (NHL) (ClinicalTrials.gov Identifier: NCT04074330).

Altered glycosylation in lymphoma contributes to metastasis and tumor growth [335]. Studies applying lectin histochemistry revealed that the loss of Phaseolus vulgaris lectin (L-PHA)-reactive oligosaccharides and their sialyation on the cell surface is associated with a poor prognosis in Burkitt’s lymphoma and DLBCL [336,337]. Using glycosylation inhibitors, sialyation of surface glycans was shown to weaken cell adhesion to extracellular matrix proteins, such as galectin-1, and promote tumor cell migration and invasiveness in human Burkitt’s lymphoma cell line and a human ALCL cell line [338]. Therefore, cell surface sialyation represents a potential target to prevent lymphoma cell invasion. Somatic mutations insert N-glycosylation sites in the immunoglobulin gene in follicular lymphoma (FL) and other germinal center-associated lymphomas [339]. Lectin binding to the oligosaccharides attached to these sites promotes survival and proliferation of tumor cells [340].

Epigenetics contribute to lymphomagenesis often due to mutations in histone modifier genes resulting in aberrant histone modifications. MS-based and immuno-based analysis has identified altered enzymatic activity of acetyltransferases, HDACs, methyltransferases and demethylases in lymphomas [314,315,316,341]. Inactivating mutations of the histone and non-histone acetyltransferase, CREBBP, have been observed in B- and T-cell lymphomas [342,343]. Using immune-based strategies, CREBBP was identified as a haploinsufficient tumor suppressor in FL and DLBCL [314]. MS-based methods identified PRMT5 as a crucial regulator of GC formation through the methylation of BCL6, a protein whose expression is often dysregulated in GC-derived B-cell lymphomas [316]. Interestingly, BCL6 recruitment of the demethylase, LSD1, is also required for GC formation and enhances BCL6-associated lymphomagenesis [341]. Chromatin immunoprecipitation, MS and immunoblot analysis identified a mechanism of DOT1L-mediated transcriptional regulation in HDAC1-deficient thymic lymphoma via PTM crosstalk between acetylation, methylation, and ubiquitination [315].

## 9. Conclusions

The adaptation and fine tuning of conventional proteomic techniques for use in the analysis of PTMs have seen significant progress in recent decades. The widespread availability of highly sensitive equipment capable of high-throughput analyses has led to breakthroughs in cancer research and the application of these techniques in blood cancer research is limitless. The analysis of PTMs, thus far, has opened avenues for translational research into the development of novel biomarker signatures and therapeutics to improve patient survival. As can be seen in the tables above, many studies have focused on phosphorylation and ubiquitination analysis. However, recently, the targeting of less common PTMs, such as SUMOylation, has been demonstrated as a promising approach for the treatment of hematological malignancies. Continuous improvements in the analytical power of mass spectrometry with simultaneous optimization of bioinformatic pipelines and enhanced sensitivity and specificity of PTM-specific antibodies will facilitate deeper analysis of obscure modifications that will indefinitely provide meaning to many molecular processes occurring in the cellular environment. In conclusion, the application of specialized proteomic techniques to study PTMs in blood cancer research has been the basis of many fundamental advances in the understanding and treatment of hematological malignancies. Improvements on existing analytical techniques to allow the analysis of protein modifications with extremely low stoichiometry will lead to the identification of novel PTM sites with unique molecular functions. Future applications of the proteomic techniques described in this review will enhance our overall understanding of hematological malignancies, leading to improvements in therapies and, thus, patient survival.

## Figures and Tables

**Table 1:** Current FDA-approved therapeutics for the treatment of various blood cancers that influence protein modifications.

Type of Therapeutic	Drug	PTM Affected	Type of Blood Cancer	Mechanism of Action	References
Kinase Inhibitors (KIs)	Ruxolitinib (JAKAFI^®^)	Phosphorylation	Myelofibrosis Polycythemia Vera	JAK2 inhibitor	[191,192]
	Midostaurin (RYDAPT^®^)	Phosphorylation	FLT3-mutant AML, Advanced systemic mastocytosis (AdvSM)	FLT3 inhibitor in AML. KIT inhibitor in AdvSM.	[193,194]
	Gilteritinib (XOSPATA^®^)	Phosphorylation	FLT3-mutant Acute myeloid leukemia (AML)	FLT3, ALK inhibitor	[195]
	Imatinib (GLEEVEC^®^)	Phosphorylation	Ph^+^ Chronic myeloid leukemia (CML), Ph^+^ Acute lymphoblastic leukemia (ALL), Myelodysplastic/ myeloproliferative diseases (MDS/MPD), Aggressive systemic mastocytosis (ASM), Chronic eosinophilic leukemia (CEL)	BCR-ABL inhibitor	[196]
	Dasatinib (SPRYCEL^®^)	Phosphorylation	Ph^+^ CML, Ph^+^ ALL	BCR-ABL, SRC inhibitor	[197]
	Nilotinib (TASIGNA^®^)	Phosphorylation	Ph^+^ CML	BCR-ABL inhibitor	[198]
	Bosutinib (BOSULIF^®^)	Phosphorylation	Ph^+^ CML	BCR-ABL and SRC inhibitor	[199]
	Ponatinib (ICLUSIG^®^)	Phosphorylation	CML, Ph^+^ ALL	BCR-ABL inhibitor	[200]
	Ibrutinib (IMBRUVICA^®^)	Phosphorylation	Mantle cell lymphoma (MCL), Chronic lymphocytic leukaemia (CLL) Small lymphocytic lymphoma (SLL) Waldenström’s macroglobulinemia (WM) Marginal zone lymphoma (MZL)	BTK inhibitor	[201]
	Idelalisib (ZYDELIG^®^)	Phosphorylation	CLL, SLL Follicular lymphoma (FL)	Phosphatidylinositol 3-kinase delta (PI3Kδ) inhibitor	[202]
Proteasome Inhibitors (PIs)	Bortezomib (VELCADE^®^)	Ubiquitination	MCL, Multiple myeloma (MM)	26S proteasome inhibitor	[203]
	Carfilzomib (KYPROLIS^®^)	Ubiquitination	MM	26S proteasome inhibitor	[204]
	Ixazomib (NINLARO^®^)	Ubiquitination	MM	26S proteasome inhibitor	[205]
Differentiation Therapy	All-trans retinoic acid (ATRA) (VESANOID^®^) and arsenic trioxide (TRISENOX^®^)	Sumoylation Ubiquitination	Acute promyelocytic leukemia (APL)	Sumoylation-dependent degradation of the fusion oncoprotein PML-RARα.	[206]
Immunomodulatory Drugs (IMiDs)	Lenalidomide (REVLIMID^®^)	Ubiquitination	MM, MDS, MCL, FL, MZL	Modulation of CRL4^CRBN^ E3 ubiquitin ligase activity.	[207]
	Thalidomide (THALOMID^®^)	Ubiquitination	MM	Modulation of CRL4^CRBN^ E3 ubiquitin ligase activity.	[208]
	Pomalidomide (POMALYST^®^)	Ubiquitination	MM	Modulation of CRL4^CRBN^ E3 ubiquitin ligase activity.	[208]
Histone Deacetylase Inhibitors (HDACi)	Panobinostat (FARYDAK^®^)	Acetylation	MM	Pan-HDAC inhibitor	[209]
	Vorinostat (ZOLINZA^®^)	Acetylation	Cutaneous T cell lymphoma (CTCL)	Class I, II HDAC inhibitor	[210]
	Belinostat (BELEODAQ^®^)	Acetylation	Peripheral T cell lymphoma (PTCL)	Pan-HDAC inhibitor	[211]
	Romidepsin (ISTODAX^®^)	Acetylation	CTCL, PTCL	Class I HDAC inhibitor	[212]

Abbreviations: JAK2, Janus kinase 2; KIT, proto-oncogene c-Kit; ALK, anaplastic lymphoma kinase; Ph^+^, Philadelphia chromosome positive; BCR-ABL, breakpoint cluster region-proto-oncogene tyrosine-protein kinase; SRC, proto-oncogene c-Src; BTK, Bruton’s tyrosine kinase; PML-RARα, promyelocytic leukemia/retinoic acid receptor alpha; CRL4^CRBN^, CRL4/Cereblon E3 ubiquitin ligase complex; HDAC, histone deacetylase.

**Table 2:** Recent studies utilizing proteomic techniques to analyze PTMs in multiple myeloma.

PTM Analyzed	Proteomic Technique	Main Finding	Reference
Phosphorylation	Western blot analysis	Myeloid-derived suppressor cells (MDSCs) drive enhanced phosphorylation of AMPK, promoting MM cell survival	[215]
Phosphorylation	Western blot analysis	PRL-3 aberrantly phosphorylates STAT3 through SHP-2 repression, leading to constant activation of STAT3	[216]
Phosphorylation	Trypsin digestion, IMAC phosphopeptide enrichment, LC–MS/MS, Western blot analysis	Elucidated signaling dynamics in MM to aid precision medicine	[217]
Phosphorylation	Trypsin digestion, IMAC phosphopeptide enrichment, LC–MS/MS, Western blot analysis	Bone marrow stromal cells stimulate the activation, through phosphorylation, of JAK/STAT signaling. Tofacitinib reverses BMSC-mediated proliferation of MM cells	[218]
Phosphorylation	SILAC labeling, trypsin digestion, SCX chromatography, IMAC phosphopeptide enrichment, phosphotyrosine immunoprecipitation, LC–MS/MS	MM cells treated with imatinib show inhibition of kinase activity due to RNA processing and a decrease in lipid biosynthesis	[219]
Ubiquitination	Human influenza hemagglutinin (HA)-tagged ubiquitin, immunoprecipitation, Western blot analysis	Destabilization of NEK2, via its ubiquitination, reduces MM cell growth and overcomes resistance to proteasome inhibitors	[220]
Ubiquitination	Immunoprecipitation, immunoblot analysis	USP15 inhibits ubiquitination and degradation of NF-κBp65 which in turn promotes USP15 expression resulting in a feedback loop enhancing MM survival	[221]
Ubiquitination	Immunoprecipitation, SDS-PAGE, trypsin digestion, LC–MS/MS	Identification of 73 ubiquitination sites on 52 ubiquitinated proteins in human MM U266 cells	[222]
SUMOylation	Immunoprecipitation, Western blot analysis	Downregulation of SENP2 increases IκBα sumoylation which activates NF-κB, leading to bortezomib resistance	[223]
SUMOylation	Cell culture, transfection, SDS-PAGE, Western blot analysis, chemiluminescence, co-immunoprecipitation	Identification of a sumoylation signature in MM that is associated with adverse clinical outcome	[224]
N-glycosylation	HILIC-solid phase extraction, MALDI–TOF–MS	Analysis of serum protein N-glycosylation in MM revealed a correlation between N-glycosylation marks and ISS stages	[225]
Glycosylation (Sialylation)	Lectin histochemistry, lectins: Sambucus Nigra (SNA), Peanut Agglutinin (PNA), Maackia Amurensis Lectin II (MALII)	Inhibition of sialylation prevents MM cell interactions with E-selectin, MADCAM1 and VCAM1 restricting the access of tumor cells to the protective BM microenvironment	[226]
Acetylation	Site directed mutagenesis, SDS-PAGE, immunoblot analysis	Inhibition of HDAC3 and DNMT1 reduces survival of MM cells	[227]
Acetylation	SDS-PAGE, immunoblot analysis	FDA-approved panobinostat increases acetylation of H3K9 resulting in IRF4 inhibition and MM cell apoptosis	[228]
Methylation	SDS-PAGE, immunoblot analysis	KDM6B, independent of demethylase activity, upregulates MAPK signaling, leading to MM survival and proliferation	[229]
Methylation Acetylation	Isotopic labeling, -Multiple reactionmonitoring based LC–MS/MS, label-free quantification	Quantification of histone PTM marks in MM cell line	[230]

Abbreviations: AMPK, 5’ adenosine monophosphate-activated protein kinase; MM, multiple myeloma; PRL-3, phosphatase of regenerating liver 3; STAT3, signal transducer and activator of transcription 3; SHP-2, SH2 domain-containing protein tyrosine phosphatase-2; IMAC, immobilized metal ion affinity chromatography; LC-MS/MS, liquid chromatography- tandem mass spectrometry; BMSC, bone marrow stromal cells; NEK2, NIMA related kinase 2; SILAC, stable isotopic labelling of amino acids in cell culture; SCX, strong cation exchange chromatography; USP15, Ubiquitin carboxyl-terminal hydrolase 15; NF-κBp65, transcription factor p65; SDS-PAGE, sodium dodecyl sulfate polyacrylamide gel electrophoresis; SENP2, SUMO specific protease 2; IκBα, inhibitor of nuclear factor kappa B, alpha; HILIC, hydrophilic interaction liquid chromatography; MALDI–TOF–MS, matrix assisted laser desorption ionization-time of flight mass spectrometry; MADCAM1, mucosal addressin cell adhesion molecule 1; VCAM1, vascular cell adhesion molecule 1; HDAC3, histone deacetylase 3; DNMT1, DNA methyltransferase 1; H3K9, histone 3 lysine 9; IRF4, interferon regulatory factor 4; KDM6B, lysine demethylase 6B; MAPK, mitogen-activated protein kinase.

**Table 3:** Recent studies utilizing proteomic techniques to analyze PTMs in acute myeloid leukemia.

PTM Analyzed	Proteomic Technique	Main Finding	Reference
Phosphorylation	Immunoprecipitation, Western blot analysis	Phosphorylation of the oncogenic kinase PIM-1L by PKCα stimulates proliferation and growth of AML cells	[246]
Phosphorylation	Trypsin digestion, iTRAQ labeling, Fe^3+^-IMAC, RP-SAX-RP, LC–MS, Western blot analysis	Resistance to chemotherapy in AML requires the phosphorylation of transcription factor, MEF2C	[247]
Phosphorylation	SILAC, filter-aided sample preparation (FASP), LC–MS	Analysis of the impact of insulin and specific inhibitors on the phosphorylation of PI3K-Akt-mTOR pathway components in AML cells	[248]
Phosphorylation	Western blot analysis	Gilteritinib reduces phosphorylation of FLT3 and its downstream targets, improving the survival of FLT3-mutated AML mouse models	[249]
Phosphorylation	Reverse-phase protein microarray (RPPA), Western blot analysis	STAT3 inhibitor, NSC-743380, induces apoptosis in SULT1A1-expressing AML cells by inhibiting the activity of the PI3K/AKT/mTOR pathway	[250]
Ubiquitination	Site-directed mutagenesis, immunoprecipitation, Western blot analysis	Ubiquitination and degradation of CDK2 results in the differentiation of AML cells through the activation of PRDX2	[251]
Ubiquitination Phosphorylation	Western blot analysis	E3 ubiquitin ligase, TRIAD1, suppresses leukemogenesis in 11q23-AML	[252]
Ubiquitination	Immunoblot analysis, immunohistochemistry	Inhibitor of E1 ubiquitin-activating enzyme, Uba1, TAK-243 selectively decreases growth and survival of AML cells.	[253]
SUMOylation	Site-directed mutagenesis, immunoprecipitation, Western blot analysis	SUMOylation of sPRDM16, promotes AML progression and inhibits differentiation	[254]
SUMOylation	Western blot analyses	SUMOylation inhibitor, 2-D08, inhibits AML cell viability through ROS accumulation-mediated apoptosis	[255]
Phosphorylation Glycosylation	Western blot analysis	Tyrosine kinase inhibition increases surface expression of FLT3 via increased glycosylation, demonstrating therapeutic potential in combination with FLT3-directed therapy	[256]
Phosphorylation Ubiquitination Glycosylation	Immunoprecipitation, Western blot analysis	Serine/threonine kinase PIM-1 stabilizes unglycosylated FLT3-ITD, promoting the activation of STAT5 signaling	[257]
Acetylation	SILAC labeling, HPLC fractionation, immunoaffinity enrichment, LC–MS/MS	HDAC inhibitors have differential impacts on the lysine acetylome in AML cells	[258]
Acetylation Phosphorylation	Immunoprecipitation, Western blot analysis	Combination of novel HDAC inhibitor, MPT0G211, with current chemotherapeutics has anti-proliferative effects on human AML cells	[259]
Methylation	Mutagenesis, Western blot analysis	Methylation of FLT3-ITD by PRMT1 supports the persistence of FLT3-ITD^+^ AML cells	[260]

Abbreviations: PIM-1L, serine/threonine-protein kinase pim-1, isoform 2; PKCα, protein kinase C alpha; iTRAQ, isobaric tags for relative and absolute quantitation; IMAC, immobilized metal ion affinity chromatography; RP-SAX-RP, reversed phase-strong anion exchange-reversed phase; MEF2C, myocyte enhancer factor 2C; SILAC, stable isotopic labelling of amino acids in cell culture; PI3K, phosphatidylinositol 3-kinase; mTOR, mammalian target of rapamycin; FLT3, FMS-like tyrosine kinase 3; STAT, signal transducer and activator of transcription; SULT1A1, sulfotransferase 1A1; CDK2, cyclin-dependent kinase 2; PRDX2, peroxiredoxin 2; TRIAD1, two RING fingers and DRIL1; sPRDM16, short form PR domain containing 16; ROS, reactive oxygen species; HPLC, high performance liquid chromatography; HDAC, histone deacetylase; PRMT1, protein arginine N-methyltransferase 1.

**Table 4:** Recent studies utilizing proteomic techniques to analyze PTMs in myeloproliferative neoplasms.

PTM Analyzed	Proteomic Technique	Main Finding	Reference
Phosphorylation	SILAC, FASP, in-gel isoelectric focusing, HPLC fractionation, LC–MS/MS, Western blot analysis	Enhanced phosphorylation of eukaryotic initiation factor 2 α subunit (eIF2α) increases the secretion of extracellular enzymes, promoting the invasiveness of CML cells	[277]
Phosphorylation	Western blot analysis	MPNs with JAK2^V617F^ mutation evade the immune system through upregulated PD-L1 expression	[278]
Phosphorylation	Western blot analysis	Selective inhibitor of mutant JAK2 (V617F), ZT55, inhibits proliferation and induces apoptosis in HEL cell line	[279]
Phosphorylation	Western blot analysis, phospho-specific protein microarray analysis	Increased RalA, a small GTPase, promotes malignant transformation and progression in CML through the activation of Ras signaling.	[280]
Ubiquitination	Immunoblot analysis	The stem cell protein, Asrij/OCIAD1, prevents the degradation of p53, thus mediating hematopoietic stem cell quiescence and preventing uncontrolled proliferation	[281]
Phosphorylation Ubiquitination	Immunoprecipitation, immunoblot analysis	KLF4 promotes leukemogenesis through the repression of DYRK2, which mediates the activation of p53 and the degradation of c-Myc in CML-like disease in mice	[282]
Phosphorylation Ubiquitination	Immunoprecipitation, Western blot analysis, TUBE-based ubiquitin-binding assay	The loss of LZTR1 function, a mediator of Ras ubiquitination and MAPK signaling contributes to TKI resistance in BCR-ABL CML cells.	[283]
Phosphorylation Ubiquitination	Immunoprecipitation, Western blot analysis, TUBE-based ubiquitin-binding assay	Type I JAK inhibitor, but not a type II inhibitor, mediates pathogenic withdrawal signaling through the accumulation of phosphorylated JAK2 by preventing dephosphorylation and ubiquitination.	[284]
Phosphorylation SUMOylation	Immunoprecipitation, Western blot analysis	Down-modulation of the β-catenin antagonist, CBY1, in CML is induced by 14-3-3 binding and enhanced SUMOylation followed by proteasomal degradation	[285]
Phosphorylation Glycosylation	Immunoprecipitation, Western blot analysis, TMT labeling, N-glycan permethylation, MALDI–TOF–MS	Mutations in CSF3R prevents N-glycosylation, promoting ligand-independent activation of the JAK/STAT pathway	[286]
Phosphorylation Glycosylation	Immunoprecipitation, Western blot analysis, size-exclusion chromatography, MS-based analysis	Mutant calreticulin (CALR) acts as a rogue chaperone thrombopoietin receptor (TpoR), immature TpoR and mutant TpoR resulting in cytokine-independent activation and constituent JAK/STAT activation	[287]
Acetylation	Western blot analysis, immunoprecipitation, SDS-PAGE, in-gel digestion, LC–MS/MS	Activation of deacetylase Sirtuin 1 (SIRT1) restores Tet methylcytosine dioxygenase 2 (TET2) activity, disrupting the maintenance of MDS HSPCs.	[288]
Acetylation	Immunoprecipitation, Western blot analysis, LC–MS/MS	Inhibition of HDAC11 downregulates the JAK/STAT pathway and induces apoptosis in MPL^W515L^-MPN mouse model	[289]
Phosphorylation Methylation	Immunoprecipitation, Western blot analysis	Overexpression of the histone demethylase, JMJD1C, in MPNs prevents H3K9me2 and HP1α-mediated repression of NFE2 resulting in a positive feedback loop	[290]

Abbreviations: SILAC, stable isotopic labelling of amino acids in cell culture; FASP, filter aided sample preparation; HPLC, high performance liquid chromatography; CML, chronic myeloid leukemia; JAK, Janus kinase; PD-L1, programmed death-ligand 1; HEL, human erythroleukemia; RalA, Ras-related protein Ral-A; OCIAD1, ovarian cancer immunoreactive antigen domain containing 1; KLF4, krüppel-like factor 4; DYRK2, dual specificity tyrosine phosphorylation regulated kinase 2; LZTR1, leucine zipper like transcription regulator 1; TKI, tyrosine kinase inhibitor; TUBE, tandem ubiquitin binding entities; BCR-ABL, breakpoint cluster region-proto-oncogene tyrosine-protein kinase; CBY1, protein chibby homolog 1; MALDI–TOF–MS, matrix assisted laser desorption ionization-time of flight mass spectrometry; CSF3R, colony stimulating factor 3 receptor; SDS-PAGE, sodium dodecyl sulfate polyacrylamide gel electrophoresis; HSPCs, hematopoietic stem/progenitor cells; HDAC11, histone deacetylase 11; MPL, myeloproliferative leukemia virus; JMJD1C, Jumonji domain-containing protein 1C; H3K9me2, histone 3 lysine 9 dimethylation; HP1α, heterochromatin protein 1; NFE2, nuclear factor erythroid 2.

**Table 5:** Recent studies utilizing proteomic techniques to analyze PTMs in lymphomas.

PTM Analyzed	Proteomic Technique	Main Finding	Reference
Phosphorylation	Immunohistochemistry, Western blot analysis	Increased STAT3 phosphorylation promotes PD-L1 expression in NKTL	[300]
Phosphorylation	Western blot analysis, PhosphoFlow cytometry, reverse phase protein microarray, TiO_2_ enrichment, LC–MS/MS	The dual PI3K/mTOR inhibitor, PQR309, demonstrates anti-cancer activity alone and in combination with other therapies in preclinical lymphoma models	[301]
Phosphorylation	Immunohistochemistry, Western blot analysis, LC–MS/MS	Phosphorylation of SOCS1 by Src family kinases inhibits SOCS1-p53-mediated senescence	[308]
Phosphorylation	Trypsin digestion, TiO_2_ enrichment, tyrosine-phosphorylated peptide immunoprecipitation, LC–MS/MS, Western blot analysis	CSF1R expression is altered in many T cell lymphomas. CSF1R activation by CSF1 leads to phosphorylation and activation of downstream PI3K/AKT/mTOR signaling, thus promoting proliferation and survival	[302]
Ubiquitination	Site-directed mutagenesis, immunoprecipitation, Western blot analysis, TUBE assay	The ubiquitin ligases cIAP1 and cIAP2 promote oncogenic BCR signaling and represent the potential of SMAC mimetics for the treatment of ABC DLBCL	[309]
Ubiquitination	Western blot analysis	Enhanced expression of TRIM11 activates β-catenin signaling via Axin1 ubiquitination and degradation, thus promoting lymphomagenesis	[310]
SUMOylation	Immunoprecipitation, Western blot analysis, in vitro SUMOylation assay, site-directed mutagenesis	Dysregulated SUMOylation in NPM-ALK^+^ T-cell lymphoma stabilizes the NPM-ALK fusion protein resulting in its accumulation promoting carcinogenesis	[311]
SUMOylation	Western blot analysis	Epstein-Barr Virus LMP1 enhances SUMOylation through interaction with the SUMO E2-conjugating enzyme, Ubc9, in LMP1-positive lymphomas	[312]
Glycosylation	Western blot analysis, glycoprotein deglycosylation assay, GC-MS	Overexpression of the glycosyltransferase, GLT1D1, is a poor prognostic marker that enhances PD-L1 glycosylation promoting tumor growth in B cell NHL	[313]
Acetylation	Western blot analysis	The tumor suppressing acetyltransferase, CREBBP, is haploinsufficient in GC-derived B cell NHL	[314]
Acetylation Methylation Ubiquitination	Western blot analysis, nanoLC-MRM, MS/MS	HDAC1-deficient thymic lymphomas show increased H3K79 methylation and demonstrate sensitivity to a DOT1L inhibitor	[315]
Methylation	Immunoprecipitation, Western blot analysis, in vitro methyltransferase assay, SDS-PAGE, trypsin digestion, LC–MS/MS	PRMT5-mediated regulation of BCL6, via methylation, is required for germinal center formation. Dual inhibition of PRMT5 and BCL6 suppresses DLBCL proliferation	[316]

Abbreviations: STAT, signal transducer and activator of transcription; PD-L1, programmed death-ligand 1; NKTL, natural killer T cell lymphoma; PI3K, phosphatidylinositol 3-kinase; mTOR, mammalian target of rapamycin; SOCS, suppressor of cytokine signalling; CSF1R, colony stimulating factor 1 receptor; CSF, colony stimulating factor; cIAP, cellular inhibitor of apoptosis protein; BCR, B cell receptor; SMAC, second mitochondria-derived activator of caspase; ABC DLBCL, activated B-cell diffuse large B cell lymphoma; TRIM11, tripartite motif containing 11; NPM-ALK, nucleophosmin-anaplastic lymphoma kinase; LMP1, latent membrane protein 1; GC/MS, gas chromatography–mass spectrometry; GLT1D1, glycosyltransferase 1 domain-containing protein 1; NHL, non-Hodgkin’s lymphoma; CREBBP, CREB binding protein; GC, germinal center; HDAC, histone deacetylase; H3K79, histone 3 lysine 79; DOT1L, disruptor of telomeric silencing 1-like; MRM, multiple reaction monitoring; PRMT5, protein arginine methyltransferase 5; BCL6, B cell lymphoma 6; SDS-PAGE, sodium dodecyl sulfate polyacrylamide gel electrophoresis.
